# Behavioural and neurodevelopmental impairment at school age following necrotising enterocolitis in the newborn period

**DOI:** 10.1371/journal.pone.0215220

**Published:** 2019-04-11

**Authors:** Mathias Lühr Hansen, Ida Voss Jensen, Rasmus Gregersen, Sandra Meinich Juhl, Gorm Greisen

**Affiliations:** Department of Neonatology, Rigshospitalet, Copenhagen, Denmark; Children's Hospital Boston, UNITED STATES

## Abstract

**Aim:**

The aim of this study was to evaluate long-term behavioural and neurodevelopmental complications of neonatal necrotizing enterocolitis at school age.

**Method:**

This was a historic cohort study comparing all surviving children born in Denmark between 1st of January 2002 and 31st of December 2011 with a diagnosis of necrotizing enterocolitis to a group of children without necrotizing enterocolitis, but same gestational age, birth weight and year of birth. Outcomes were investigated through a parental questionnaire. The primary outcome was the Strength and Difficulties Questionnaire score and secondary outcomes were cerebral palsy and impaired head growth.

**Results:**

Response rates were 50% (163 of 328) and 36% (237 of 652) among children with and without necrotizing enterocolitis, respectively. There was a higher rate of abnormal Strength and Difficulties score (23.9 versus 17.8%), moderate/severe cerebral palsy (3.1 versus 0.9%) and small head circumference for age (11.7 versus 7.2%) among children with necrotizing enterocolitis. However, these differences were all statistically insignificant and did not change significantly by adjustment for potential confounders.

**Conclusion:**

To our knowledge, this study includes the largest cohort of necrotizing enterocolitis children evaluated for possible long-term complications at school age. The increased risks of behavioural- and neurodevelopmental impairments were statistically insignificant, moderate in magnitude and may be of little clinical importance for management in the neonatal period or when planning follow-up.

## Introduction

Necrotizing enterocolitis (NEC) is a severe gastrointestinal emergency seen among premature infants in the NICU, with the majority weighing less than 1500 grams [1]. In this subgroup, NEC has a reported incidence varying from 5% to 12% [2–4]. Recent advances in neonatal care have increased the survival of premature infants, thereby increasing the population at risk of NEC. Despite progress in neonatal care, NEC mortality remains high (30–50%) with the worst prognosis in infants requiring surgical treatment [5–8]. Systemic circulatory compromise and excessive inflammation caused by NEC may lead to multiple organ failure, including brain injury. The majority of today’s research have focused on the risk factors related to NEC and the short-term outcomes during hospital admission. Fewer studies have focused on the long-term consequences of brain injury following an episode of NEC. As numbers of NEC survivors are increasing, it is important to clarify the possible sequelae later in life following NEC.

The aim of this study was to evaluate the behavioural and neurodevelopmental complications of neonatal necrotizing enterocolitis at school age.

## Methods

### Study design and population

This was a nationwide historic cohort study. We included all children born in Denmark between 1^st^ of January 2002 and 31^st^ of December 2011 with an ICD-10 diagnosis of NEC (DP77.9) and surviving until follow-up at 1^st^ of March 2017. These children were compared to a group of children with no history of NEC, but same gestational age, year of birth and birthweight. For each child with NEC, we included two children without NEC. The children were identified from The Medical Birth Registry and the presence of NEC was recorded from the Danish National Patient Registry [9]. Matching criteria were chosen to balance prematurity, degree of intrauterine growth restriction and age at follow-up between the groups. The same cohort was used to study gastrointestinal sequalae and growth impairment among NEC children [Hansen et al, unpublished].

### Outcomes

Outcomes were investigated through parental questionnaires, sent by mail to the parents. Parents were suggested to involve the children in the assessments if relevant. The questions explored behaviour using the Strengths and Difficulties Questionnaire (SDQ) [10] motor impairment using the Gross Motor Function Classification for cerebral palsy (CP) [11], and using questions regarding vision and hearing. Furthermore, the parents also received information about the study, instructions and a measure tape to examine head circumference and height of their child. Parents and children were not contacted prior to receiving the questionnaire. It was stated in the study information, that by returning the questionnaire, the parents gave their consent to inclusion of their child in the study. consent was given to be included in the study. Permission to send out parental questionnaires was obtained from the National Board of Health (j.nr. FSEID-00002564) and permission to store and acquire the information was granted from the Danish Data Protection Agency (j.nr. 2012-58-0004). According to Danish law, Act on Research Ethics Review of Health Research Projects, section 14 paragraph 2, permission from the Research Ethics Committee was not required (j.nr. H-18053160). The protocol is available from clinicaltrials.gov: Identifier NCT03091907.

Children with NEC were compared to children without NEC, on primary and secondary outcomes. Furthermore, we report exploratory outcomes, but these were not tested for significance. The primary outcome was defined as an abnormal or borderline score (≥14) for the ‘total difficulties score’ in the SDQ (range 0 to 40). Secondary outcomes included the following outcomes; cerebral palsy dichotomized into non/mild cerebral palsy (scoring lower than III) and moderate/severe cerebral palsy (scoring III or higher) in the Gross Motor Function Classification. Head circumference was analysed both as standard deviation (SD)-score (age and gender standardized head circumference) calculated from Growth XP (version 2.5, PC-PAL, Denmark), and as ‘low head circumference-for-age’ (head circumference SD-score <-2 SD). Exploratory outcomes consisted of visual impairment (being blind or having impaired vision despite correcting with glasses or contact lenses) and hearing impairment (being deaf and/or using hearing aids).

### Explanatory variables

Explanatory variables were either present at birth or occurred within the first year of life. Variables present at birth were extracted from the Danish Medical Birth Registry and consisted of gestational age (GA, in completed weeks), gender, birthweight, head circumference, birth length, caesarean section and multiple births. Variables occurring within the first year of life were identified from the Danish National Patient Registry diagnosis codes (by the Danish modification of ICD-10) or procedure codes (NOMESCO Classification of Surgical Procedures) Table 1.

**Table 1:** Definition of variables used in present study. Diagnosis codes by Danish modification of ICD-10 and procedure codes by NOMESCO Classification of Surgical Procedures, translated from the Danish Health Authority Classification System. ^x^ = subgroups included.

Explanatory variable	Code definition
Sepsis	DP36^x^ “*Bacterial sepsis of the newborn*”
Congenital heart disease	DQ2^x^ “*Congenital anomalities of circulatory organs*”
Severe brain injury	DP522 “*Non-traumatic intraventricular haemorrhage*, *grade 3*”
	DP524 “*Non-traumatic intracerebral haemorrhage of the newborn*”
	DP911 “*Acquired periventricular cysts of the new born*”
	DP912A “*Periventricular cerebral leukomalacia of the newborn*”
	DG91 “*Hydrocephalus*”
	DQ03^x^ “*Congenital hydrocephalus*”
Non-traumatic brain haemorrhage	DP52^x^ “*Non-traumatic haemorrhage of the newborn*”
Retinopathy of prematurity	DH351 “*Retinopathy of prematurity*”
Bronchopulmonary dysplasia	DP271^x^ “*Bronchopulmonary dysplasia arising in the perinatal-period*”
Respiratory distress syndrome	DP22^x^ “*Respiratory distress of the newborn*”
Abdominal surgery	KJA^x^ “*Procedures on abdominal wall*, *peritoneum*, *mesentery*, *or omentum*”
	KJD^x^ “*Procedures on stomach and duodenum*”
	KJF^x^ “*Procedure on the small and large intestines*”
Respirator treatment	BGDA0 “*Respirator treatment*” BGDA00 “*PEEP-treatment with respirator*”
Surfactant therapy	BGHF^x^ “*Treatment with surfactant*”
Use of parental nutrition	BUAL1 “*Complete parenteral nutrition*”

Due to a low occurrence of severe intraventricular haemorrhage, periventricular leukomalacia, and hydrocephalus, we combined them into one parameter named ‘severe brain injury’. Data regarding birth and perinatal history was obtained to adjust for possible confounders in the statistical analysis.

### Sample size and power calculation

The sample size was determined by the available number of children with NEC, i.e. children at school age in Denmark with a previous diagnosis of NEC and by the response rate. We estimated that 200 children with NEC and 300 children without NEC would respond and hereby be included in our study. An indicative power calculation assuming 30% prevalence of abnormal/borderline SDQ-score among children with NEC and 20% among children without NEC [12] showed that this study could detect a difference between children with previous NEC and children without previous NEC, with 72% power at a 5% significance level.

### Statistical analyses and model building strategy

Medians and ranges were used to report continues variables, and frequencies along with the proportion of missing values, for categorical variables. To identify potential dropout, we used χ2-test, ANOVA, or Kruskall-Wallis test as appropriate, to compare responders and non-responders across children with and without NEC. Univariable analyses were performed for the primary and all secondary outcome by χ2-test, Fisher’s Exact test or t-test, as appropriate comparing children with NEC to children without NEC. The primary outcome of behavioural impairment along with the secondary outcomes of cerebral palsy, head circumference and low-head-circumference-for-age underwent multivariable analyses. In the multivariable analyses we included NEC, gender, GA, congenital heart disease, severe brain injury, surfactant therapy, and respirator treatment, under suspicion of potential confounding. These variables were chosen since they were likely to influence the risk of impairments due to brain injury. Initially, all multivariable outcomes were analysed with each chosen explanatory variable univariate, by χ2-test, Fisher’s Exact test, t-test or linear regression, as appropriate. All explanatory variables associated with any of the four outcomes with p<0.20, were included in multivariable analyses for all four outcomes. Finally, we conducted backwards elimination to p<0.20 for all outcomes, separately. We did not investigate effect-modification. For the primary outcome, a p-value of <0.05 was considered significant. With consideration to multiple testing, we chose a p-value of <0.01 as significant for secondary outcomes. No post-hoc corrections were made. Statistical analyses were performed using SAS Statistical Software (Version 9.4, SAS Institute Inc, North Carolina, USA).

### Ad hoc analysis

Since children with surgically managed NEC tend to be more ill than those medically managed, we decided to perform an explorative ad hoc subgroup analysis for the SDQ outcome, by dividing children with NEC into surgical or medical treated and comparing these two subgroups individually to children without NEC.

## Results

Of all surviving children born in Denmark between 1^st^ of January 2002 and 31^st^ of December 2011, 328 had been registered with the ICD-10 diagnosis of NEC. Of these, 163 (49.7%) responded and were included in the group of children with NEC. Through the Danish National Patient Registry, we identified 652 children fulfilling the matching criteria. Of these, 237 (36.7%) responded and thus were included in the group of children without NEC.

Birth demographics were similar between children with and without NEC. Children with NEC had more often been diagnosed with congenital heart disease (primarily persistent arterial duct), respiratory distress syndrome, sepsis and ROP within the first year of life. The use of surfactant therapy, respirator treatment, gastrointestinal surgery and parenteral nutrition had also been more frequent in the children with NEC Table 2.

**Table 2:** Baseline characteristics for the children with NEC and children without NEC.

	Children with NEC (n = 328)	Children without NEC (n = 652)
**Birth demographics**		
Age at follow-up (years)	10.5 (5.6–15.6)	10.5 (5.6–15.6)
Weight (gram)	1442.5 (470–4810)	1454.7 (483–4850)
	1 (0.3) missing	26 (4.0) missing
Gestational age (weeks)	29.7 (23.0–42.0)	29.8 (23.0–42.0)
	0 missing	18 (2.8) missing
Length (cm)	39.9 (20.0–56.0)	40,5 (23.0–58.0)
	73 (22.3) missing	159 (24.4) missing
Head circumference (cm)	28.1 (20.0–38.0)	28,5 (16.0–41.0)
	112 (34.1) missing	227 (41.7) missing
Girl	151 (46.0)	319 (48.9)
Sectio	230 (70.1)	401 (61.5)
Multiple birth	103 (31.4)	232 (35.6)
**Diseases within first year of life**		
Sepsis	190 (57.9)	231 (35.4)
Congenital heart disease	117 (35.7)	161 (24.7)
Severe brain injury	19 (5.8)	31 (4.8)
Severe IVH	11 (3.4)	19 (2.9)
Nontraumatic brain haemorrhage	54 (16.5)	81 (12.4)
Periventricular leukomalacia	9 (2.7)	7 (1.1)
Hydrocephalus	4 (1.2)	16 (2.5)
ROP	68 (20.7)	84 (12.8)
BPD	78 (23.8)	151 (23.2)
RDS	232 (70.7)	406 (62.3)
**Treatment within first year of life**		
Abdominal surgery	166 (50.6)	38 (5.8)
Respirator	230 (70.1)	182 (27.9)
Surfactant	112 (34.2)	200 (30.7)
Perenteral nutrition	250 (76.2)	265 (40.6)

Mean (min-max) for continuous variables, number (%) for categorical. IVH (intraventricular hemorrhage), ROP (retinopathy of prematurity), BPD (bronchopulmonary dysplasia), RDS (respiratory distress syndrome), NEC (necrotizing enterocolitis).

There was a significant difference between responders and non-responders in the frequency of sepsis (p<0.0001), congenital heart disease (p = 0.0001), ROP (p = 0.0006) and RDS (p = 0.0013). The overall pattern was that non-responding children with NEC tended to have had a higher rate of diseases within first year of life compared to responding children with NEC and the opposite being true for the children without NEC, with responders being more ill than non-responders Table 3.

**Table 3:** Baseline characteristics for responders and non-responders in children with NEC and children without NEC.

	Children with NEC (n = 328)		Children without NEC (n = 652)		P-value
	Responders: 163 (49.7%)	Non responders: 165 (50.3%)	Responders: 237 (36.4%)	Non responders: 415 (63.6%)	< 0.0001
Age at follow-up (years)	10.5 (5.7–15.5)	10.6 (5.6–15.6)	10.5 (5.6–15.5)	10.6 (5.6–15.6)	0.99
**Birth demographics**					
Weight (gram)	1432 (480–4810)	1413 (470–4310)	1329 (483–4745)	1527 (530–4850)	0.12^a^
Gestational age (weeks)	30 (23–42)	30 (23–42)	29 (23–42)	30 (24–42)	0.14 ^a^
Length (cm)	40 (20–56)	39 (23–56)	40 (23–55)	41 (25–58)	0.07 ^a^
Head circumference (cm)	28 (20–38)	28 (20–37)	28 (20–38)	29 (16–41)	0.051 ^a^
Female gender	80 (49.1%)	71 (43.0%)	112 (47.3%)	207 (49.9%)	0.50
Sectio	111 (68.1%)	119 (72.1%)	145 (61.2%)	256 (61.7%)	0.53
Multiple birth	54 (33.1%)	49 (29.7%)	81 (34.2%)	151 (36.4%)	0.48
**Diseases in the neonatal period / within first year of life**					
RDS	111 (68.1%)	121 (73.3%)	164 (69.2%)	242 (58.3%)	0.0013
Sepsis	91 (55.8%)	99 (60%)	88 (37.1%)	143 (34.5%)	< 0.0001
Congenital heart disease	62 (38.0%)	55 (33.3%)	73 (30.8%)	88 (21.2%)	0.0001
Severe IVH	4 (2.5%)	7 (4.2%)	6 (2.5%)	13 (3.1%)	0.75
Nontraumatic brain haemorrhage	24 (14.7%)	30 (18.2%)	29 (12.2%)	52 (12.5%)	0.28
Periventricular leukomalacia	3 (1.8%)	6 (3.6%)	3 (1.3%)	4 (1.0%)	0.14
Hydrocephalus	2 (1.2%)	2 (1.2%)	6 (2.5%)	10 (2.4%)	0.64
Severe brain injury	7 (4.3%)	12 (7.3%)	11 (4.6%)	20 (4.8%)	0.57
ROP	27 (16.6%)	41 (24.9%)	38 (16.0%)	46 (11.1%)	0.0006
BPD	30 (18.4%)	48 (29.1%)	55 (23.2%)	96 (23.1%)	0.15
**Treatment within first year of life**					
Surfactant	50 (30.7%)	62 (37.6%)	78 (32.9%)	122 (29.4%)	0.28
Respirator	110 (67.5%)	120 (72.7%)	76 (32.1%)	106 (25.4%)	< 0.0001
Parenteral nutrition	123 (75.5%)	127 (77.0%)	103 (43.5%)	162 (39.0%)	< 0.0001
NEC related gastrointestinal surgery	91 (55.8%)	75 (45.5%)	17 (7.2%)	21 (5.1%)	< 0.0001

Continuous variables displayed by mean (min-max) and categorical by n (%). P-value calculated from Chi-square (categorical data), and ANOVA or Kruskall-Wallis test ^a^ (continuous data). IVH (intraventricular haemorrhage), ROP (retinopathy of prematurity), BPD (bronchopulmonary dysplasia)

### Behavioural impairment

Results from the parents-reported SDQ showed an increased risk of abnormal/borderline SDQ in children with NEC compared to children without NEC (23.9% versus 17.8%) Table 4.

**Table 4:** Occurrence of primary and secondary outcome and their association with NEC in univariable analysis.

	Children with NEC (n = 163)	Children without NEC (n = 237)	Parameter estimate (95% CI)	P-value
Borderline/abnormal SDQ-score	39 (23.9)	42 (17.8)	1.45 (0.89–2.37)	0.13
	Missing: 0	Missing: 1		
Cerebral palsy	5 (3.1)	2 (0.9)	3.67 (0.70–19.2)	0.13 ^a^
	Missing: 0	Missing: 3		
Head circumference (SDS)	-0.47 (1.16)	-0.31 (1.19)	-0.15 (-0.39 0.08)	0.20
	Missing: 2	Missing: 4		
Small head circumference-for-age	19 (11.8)	17 (7.3)	1.70 (0.85–3.38)	0.12
	Missing: 2	Missing: 4		

Displayed as n (%) for binary variables and mean (SD) for continuous variables. Parameter estimates as odds ratio for binary variables and mean difference for continuous. Analysis by Chi-Square or T-test, unless specified otherwise.

^a^ = Fisher’s Exact Test

SDS (standard deviation score)

Median SDQ scores were 9.1 and 8.0 respectively. In univariable analysis for abnormal/borderline SDQ score, point estimate of OR comparing children with NEC to children without NEC was greater than one but statistically insignificant (OR 1.45, 95% CI 0.89 to 2.37). Adjustment for possible confounders in the multivariable analysis with backwards elimination did not alter the results (OR 1.41, 95% CI 0.86 to 2.32) Table 5.

**Table 5:** Univariate and multivariate analyses for the outcomes SDQ-score, cerebral palsy and head circumference.

	Univariate analysis		Initial multivariate analysis (full model)		Multivariate analysis after backwards elimination	
Outcome	OR (95% CI)	P-value	OR (95% CI)	P-value	OR (95% CI)	P-value
Borderline/abnormal SDQ-score	1.45 (0.89–2.37)	0.13	1.32 (0.76–2.30)	0.32	1.41 (0.86–2.32)	0.18
Cerebral palsy	3.67 (0.70–19.2)	0.13 ^a^	13.71 (1.15–163.39)	0.03	4.1 (0.74–22.84)	0.11
Small head circumference-for-age	1.70 (0.85–3.38)	0.13	1.81 (0.82–4.02)	0.14	1.91 (0.92–3.96)	0.08

Multivariate analyses were adjusted for all explanatory variables with p < 0.20 for any of the three outcomes. Backwards elimination by p-value to all variables in the model had p-value < 0.20

^a^ = Fisher’s Exact Test

OR (odds ratio)

The explorative ad hoc analysis showed no significant association between abnormal/borderline SDQ score and surgically or medically managed NEC, respectively (OR 1.65, 95% CI 0.93 to 2.94 and OR 1.22, 95% CI 0.63 to 2.35). However, more children with surgically managed NEC had abnormal/borderline SDQ score (26.4% compared to children with medically managed NEC (20.8%).

### Other impairments

Moderate/severe cerebral palsy was more frequently reported in NEC children (3.1% vs 0.9%) who also had a higher frequency of small head circumference-for-age (11.8% vs 7.3%). Head circumference standard-deviation scores were also lower in children with NEC, with a mean of -0.47 SDS compared to -0.31 SDS in children without NEC. Furthermore, vision- and hearing impairment were more frequent in children with NEC (3.7% vs 2.6% and 3.1% vs 1.3%, respectively). In univariable and multivariable analyses of cerebral palsy and head circumference, all point estimates of OR comparing children with NEC to children without NEC, were greater than one but statistically insignificant (cerebral palsy OR 3.67, 95% CI 0.7 to 19.2 and OR 4.1, 95% CI 0.74 to 22.84, small head circumference-for-age OR 1.70, 95% CI 0.85 to 3.38 and OR 1.91, 95% CI 0.92 to 3.96, head circumference standard deviation-score mean diff -0.15, 95% CI -0.39 to 0.08 and -0.36, 95% CI -0.86 to 0.13). After backwards elimination in the multivariable model, the explanatory variable “severe brain injury” was significantly associated with cerebral palsy (p = 0.0003) as well as with the continuous (p = 0.03) and dichotomized (p<0.0001) measure of head circumference. Female gender was also significantly associated with head circumference standard deviation-score (p = 0.01).

## Discussion

This study suggests that the negative consequences of the NEC-associated brain injury detected in early childhood may not be important at school age at the population level. The primary outcome was behavioural impairment, which revealed an increased risk, though moderate of magnitude and not statistically significant. The same trend was found in the analyses of cerebral palsy and head circumference.

### Behaviour

Behavioural difficulties seem to be of practical importance for children at school age. Two other studies have investigated this in children with NEC Table 6.

**Table 6:** Characteristics of behavioural and cerebral palsy studies.

Author	Year	Age at follow up (years)	Birthweight (mean, g)		Outcome							
					Behavioural difficulties				Cerebral palsy			
			Children with NEC	Children without	NEC n/N (%)	No NEC n/N (%)	OR (95%CI)	RD (%)	NEC n/N (%)	No NEC n/N (%)	OR (95% CI)	RD (%)
Rees ^a^	2006	<3	n/a	n/a	n/a	n/a	n/a	n/a	79/393 (20.1)	590/3984 (14.8)	1.55 (1.19;2.03)	5.3
Soraisham	2006	3	997	981	n/a	n/a	n/a	n/a	8/46 (17.4)	8/100 (8)	2.2 (0.7;6.9)	9.4
Roze	2011	9	1100(medical) 1415(surgical)	1220	15/50 (30)	11/31 (35.5)	0.78 ^b^ (0.30;2.02)	-5.5	n/a	n/a	n/a	n/a
Dilli	2012	<3	1065	1240	n/a	n/a	n/a	n/a	2/20 (10)	4/40 (10)	1.00^b^ (0.17;5.98)	0
Pike	2012	7	n/a	n/a	33/114 (28.9)	1364/6109 (22.3)	1.38 (0.92;2.08)	6.6	8/118 (6.8)	159/6316 (2.5)	2.77 (1.33;5.77)	4.3
Wadhawan	2014	<3	736	765	n/a	n/a	n/a	n/a	43/191 (22.5)	300/5433 (5.5)	4.97 (3.47;7.12	17
Current study	2018	10.5	1432	1329	39/163(23.9)	42/236 (17.8)	1.45 (0.89;2.37)	6.1	5/163 (3.1)	2/234 (0.9)	3.67 (0.70;19.2)	2.2

^a^ meta-analysis of five studies from 1989 to 2005. Three with BW <1500g, one with 995g mean BW and one with BW<1000g

^b^ Calculated from reported data.

n/a, data is not available in respective studies.

In line with our findings, a trend towards increased behavioural difficulties was found as measured by SDQ among 114 children with NEC at seven years of age compared to 6,109 unmatched controls (28.9 vs 22.5%) [12]. The second study reported less behavioural difficulties, measured by the parental Child Behavioural Checklist, in 32 NEC and 20 spontaneous intestinal perforation (SIP) cases at nine years of age compared to 31 matched controls (28.8 vs 35.5%) [13]. However, inclusion of SIP cases, makes a direct comparison to our results difficult, since SIP is typically a less severe disease than NEC, with higher survival rate and possibly better neurodevelopmental outcomes [14].

### Cerebral palsy and other neurodevelopmental outcomes

Our study showed a trend towards an increased risk of moderate-to-severe cerebral palsy at school age in the NEC group but with low event rates in both groups (Table 6). Only the ORACLE children study has studied neurodevelopmental outcome at school age [12]. They evaluated the association at seven years of life between NEC and cerebral palsy (6.8 vs 2.5%), seizures (11.0 vs 7.3%) and ADHD (15.3 vs 7.3%). As in our study, all outcomes had a point estimate of OR greater than one [12].

Others have evaluated neurodevelopmental outcomes in pre-school aged children. At a median follow-up time of 20 months, five studies (393 NEC children in total) were included in a meta-analysis of cerebral palsy (20.1 vs 14.8%). The same study reported outcomes for visual impairment (3.4 vs 1.3%) among 296 NEC children from three studies, cognitive impairment (36.0 vs 24.0%) among 369 NEC children from seven studies and psychomotor impairment (35.1 vs 23.2%) among 328 NEC children from five studies, all statistically significantly associated with NEC [15]. At last, we found three short-term studies that were not included in the meta-analysis. Two of the studies reported a statistically significant increased risk of cerebral palsy among children with a birthweight <1000g that were surgically treated for NEC compared to controls (22.5 vs 5.5%) (14) and among children with a birthweight <1250g (17.4 vs 8%) [16]. The last of the three studies included infants <1500 g and found no trend towards increased risk of cerebral palsy in NEC children (10 vs 10%) [17]. Though these studies have a shorter follow up time, it is reasonable to assume that the findings of increased risk of cerebral palsy at pre-school age is comparable to our findings at school age due to the permanent and non-progressive nature of cerebral palsy.

### Head circumference

Our analysis showed a trend of higher prevalence of small head circumference-for-age at school age in children with NEC compared to children without NEC, which is coherent with our findings regarding cerebral palsy and behavioural difficulties. Head circumference has not previously been used as a neurological outcome when investigating NEC consequences later in childhood, though microcephalus is strongly associated to neurodevelopmental impairment and cerebral palsy at two years of age among preterm infants [18]. Nevertheless, six studies used head circumference as a measure of growth, all with a follow up of three years or less. Similar to our findings a study with 36 NEC children and 766 controls found a statistically significant increased risk of small head circumference-for-age among the NEC children (30 vs 13%) [19]. Another study compared medically and surgically treated NEC children to controls and found smaller head circumferences among NEC children of both groups, although the difference in head circumference was only statistically significant between surgically treated NEC children and control children [20]. Four other studies found statistically insignificant differences of head circumference between NEC children and matched controls [16,17,21,22].

We found an unexpected association between female gender and head circumference SD-scores, possibly explained by less difference of head circumference between sexes in Growth XP (version 2.5, PC-PAL, Denmark) than in other tables of reference [23].

### Strengths and weaknesses

This study stands out among studies investigating NEC consequences due to the large number of children with NEC and the long follow-up period. The rarity of NEC poses a problem to sample size; to address this problem, children from all parts of Denmark were included. Furthermore, the use of SDQ made it possible to include children with a wide age span, thus having a larger sample size available [24].

The weaknesses were that outcome measures were restricted to what could be assessed by the format of a parental questionnaire, the low response rate, and the potentially biased response. Compared to our indicative power-calculations we experienced a lower response rate, a lower occurrence of an abnormal SDQ-score, and a smaller difference between our exposure and control group than expected. This may have lowered our indicative calculated power of 72%, thus hindering reliability of negative findings and reducing our ability to make strong conclusions. Furthermore, the comparisons between the groups could have been biased by different participation in the two groups; Responding children with NEC were slightly larger and less ill in the neonatal period than non-responding children with NEC, while the responding children without NEC were smaller and more ill than the non-responding children without NEC. This could potentially reduce the estimated negative effects of NEC.

### Onset of NEC

Using registry data to identify children with NEC and create our cohort, comes with limitations in regards of onset of NEC, since these data will not be registered until discharge from hospital. Therefore, the specific time of NEC onset is uncertain and theoretically, there may have been children in our cohort who suffered from NEC after the neonatal period. In 301 of 328 children with NEC, the diagnosis of NEC was registered before six months of age. For the remaining 27, we did not have data on the date of discharge with NEC.

## Conclusion

The present data is in agreement with the literature regarding the increased risk of cerebral palsy and reduced head circumference, although our results were not statistically significant. There was a consistent trend towards an increased risk of behavioural difficulties in school age. The effects, however, appeared small to moderate in magnitude and therefore the practical importance of these findings is uncertain. It is questionable if they should lead to changes in management of NEC in the neonatal period or indicate a need for special follow-up.

## References

[1] Neu J, Walker WA. Necrotizing Enterocolitis. N Engl J Med. 2011;364:255–64. 10.1056/NEJMra1005408 21247316 PMC3628622

[2] Luig M, Lui K. Epidemiology of necrotizing enterocolitis—Part I: Changing regional trends in extremely preterm infants over 14 years. J Paediatr Child Health. 2005;41:169–73. 10.1111/j.1440-1754.2005.00582.x 15813869

[3] Hein-Nielsen AL, Petersen SM, Greisen G. Unchanged incidence of necrotising enterocolitis in a tertiary neonatal department. Dan Med J. 2015;62:A5091 26183041

[4] Yee WH, Soraisham AS, Shah VS, Aziz K, Yoon W, Lee SK, et al Incidence and Timing of Presentation of Necrotizing Enterocolitis in Preterm Infants. Pediatrics. 2012;129:298–304. 10.1542/peds.2011-2022 22271701

[5] Fitzgibbons SC, Ching Y, Yu D, Carpenter J, Kenny M, Weldon C, et al Mortality of necrotizing enterocolitis expressed by birth weight categories. J Pediatr Surg. 2009;44:1072–6. 10.1016/j.jpedsurg.2009.02.013 19524719

[6] Thyoka M, de Coppi P, Eaton S, Khoo K, Hall N, Curry J, et al Advanced Necrotizing Enterocolitis Part 1: Mortality. Eur J Pediatr Surg. 2012;22:8–12. 10.1055/s-0032-1306263 22434227

[7] Lakhoo K, Morgan RD, Thakkar H, Gupta A, Grant HW, Wagener S, et al Exploratory laparotomy in the management of confirmed necrotizing enterocolitis. Ann Pediatr Surg. 2015;11:123–6.

[8] Chacko J, Ford WDA, Haslam R. Growth and neurodevelopmental outcome in extremely-low-birth-weight infants after laparotomy. Pediatr Surg Int. 1999;15:496–9. 10.1007/s003830050648 10525908

[9] Schmidt M, Schmidt SA, Sandegaard JL, Ehrenstein V, Pedersen L, Sørensen HT. The Danish National Patient Registry: a review of content, data quality, and research potential. Clin Epidemiol. 2015;7:449–90. 10.2147/CLEP.S91125 26604824 PMC4655913

[10] Goodman R, Meltzer H, Bailey V. The Strengths and Difficulties Questionnaire: a pilot study on the validity of the self-report version. Int Rev Psychiatry. 2003;15(1–2):173–7. 10.1080/0954026021000046137 12745329

[11] Bartlett DJ, Galuppi B, Palisano RJ, Mccoy SW. Consensus classifications of gross motor, manual ability, and communication function classification systems between therapists and parents of children with cerebral palsy. Dev Med Child Neurol. 2016;58(1):98–9. 10.1111/dmcn.12933 26767662

[12] Pike K, Brocklehurst P, Jones D, Kenyon S, Salt A, Taylor D, et al Outcomes at 7 years for babies who developed neonatal necrotising enterocolitis: the ORACLE Children Study. Arch Dis Child Fetal Neonatal Ed. 2012;97:318–22. 10.1136/fetalneonatal-2011-300244 22933088

[13] Roze E, Ta BDP, Van Der Ree MH, Tanis JC, Van Braeckel KNJA, Hulscher JBF, et al Functional impairments at school age of children with necrotizing enterocolitis or spontaneous intestinal perforation. Pediatr Res. 2011;70:619–25. 10.1203/PDR.0b013e31823279b1 21857378

[14] Wadhawan R, Oh W, Hintz SR, Blakely ML, Das A, Bell EF, et al Neurodevelopmental outcomes of extremely low birth weight infants with spontaneous intestinal perforation or surgical necrotizing enterocolitis. J Perinatol. 2014;34:64–70. 10.1038/jp.2013.128 24135709 PMC3877158

[15] Rees CM, Pierro A, Eaton S. Neurodevelopmental outcomes of neonates with medically and surgically treated necrotizing enterocolitis. Arch Dis Child Fetal Neonatal Ed. 2007;92:193–8. 10.1136/adc.2006.099929 PMC2675329 16984980

[16] Soraisham AS, Amin HJ, Al-Hindi MY, Singhal N, Sauve RS. Does necrotising enterocolitis impact the neurodevelopmental and growth outcomes in preterm infants with birthweight ≤1250 g? J Paediatr Child Health. 2006;42:499–504. 10.1111/j.1440-1754.2006.00910.x 16925534

[17] Dilli D, Eras Z, Ulu HÖ, Dilmen U, Sakrucu ED. Does necrotizing enterocolitis affect growth and neurodevelopmental outcome in very low birth weight infants? Pediatr Surg Int. 2012;28:471–6. 10.1007/s00383-012-3051-4 22274546

[18] Cheong JLY, Hunt RW, Anderson PJ, Howard K, Thompson DK, Wang HX, et al Head Growth in Preterm Infants: Correlation With Magnetic Resonance Imaging and Neurodevelopmental Outcome. Pediatrics. 2008;121:1534–40. 10.1542/peds.2007-2671 18519457

[19] Walsh MC, Kliegman RM, Hack M. Severity of necrotizing enterocolitis: influence on outcome at 2 years of age. Pediatrics. 1989;84:808–14. 2797976

[20] Hintz SR. Neurodevelopmental and Growth Outcomes of Extremely Low Birth Weight Infants After Necrotizing Enterocolitis. Pediatrics. 2005;115:696–703. 10.1542/peds.2004-0569 15741374

[21] Abbasi S, Pereira GR, Johnson L, Stahl GE, Duara S, Watkins JB. Long-term assessment of growth, nutritional status, and gastrointestinal function in survivors of necrotizing enterocolitis. J Pediatr. 1984;104:550–4. 6707816 10.1016/s0022-3476(84)80545-4

[22] Sonntag J, Grimmer I, Scholz T, Stahl GE, Duara S, Watkins JB. Growth and neurodevelopmental outcome of very low birthweight infants with necrotizing enterocolitis. Acta Paediatr. 2000;89:528–32. 10852186 10.1080/080352500750027790

[23] World Health Organization. World Health Organization, growth standards head circumference-for-age [Internet]. [cited 2018 May 17]. Available from: http://www.who.int/childgrowth/standards/hc_for_age/en/

[24] Obel C, Heiervang E, Rodriguez A, Heyerdahl S, Smedje H, Sourander A, et al The strengths and difficulties questionnaire in the nordic countries. Eur Child Adolesc Psychiatry, Suppl. 2004;13. 10.1007/s00787-004-2006-2 15243784

